# Consumption

**Published:** 1872-12

**Authors:** 


### CONSUMPTION

Is the "oxidation of the exudation corpuscle." This is, perhaps, the most accurate designation of this terrible disease, meaning that it is the burning up of little particles which exude from the lungs — from the more minute blood-vessels in the lungs, in consequence of their being so full of blood, so congested, that the sides of these blood-vessels were distended so that the more fluid parts of the blood oozed through, and hardened into little tubers or drops, as gum hardens as it oozes through the bark of a tree in little droplets or rounded knobs, in size from a pin-head to a pea. These are called tubercles, and are the seeds of consumption; from them common consumption of the lungs always springs; without them it can never occur. But these droplets are not necessarily fatal; they never are unless they soften, oxidize, burn up, and eat away the lungs. They are made to soften by bad colds, or any protracted, weakening disease; hence, if persons have tubercles, they would never die of consumption if they never took a cold, and kept up good general health. A bad cold can never cause common consumption unless the seeds of it were there before; that is, tubercles. A bad cold is to tubercles what fire is to powder; explosion of the powder could never take place unless fire was applied; colds could not cause consumption unless the tubercles were there before.

Statistics show that forty-nine thousand persons die in one year in the United States from consumption, twenty-six thousand of whom are females and twenty-three thousand males, showing that more women die of the disease than men, owing, doubtless, to their in-door life, proving that "exposure" to the weather is not an element of the disease.

From the statistics given, it does not appear that any case resulted from neglected catarrh. It is to be hoped that every newspaper in the United States will make this humane announcement, to prevent its subscribers from paying away tens and hundreds of dollars to advertising physicians, in the fear that if not cured of their catarrh, consumption is a sure result, when the truth is, so far from its causing or tending to consumption, it is really a preventive, by detaining the disease in the head.

There are two kinds of tubercles: the gray or moist, called moist because they soften and eat away the lungs, and miliary, which are very thick, numerous, and hard, and yield little or no mucous or mattery expectoration. There is reason to believe that this is the kind to be benefited by living on fat meats and drinking oils, while such nutrients or foods or remedies have no beneficial effect whatever on the moist kind, that is, the ordinary form of consumptive disease, during the progress of which so much yellow matter is expectorated. Tubercles may exist in any part of the body; very frequently in the liver; sometimes in the brain, and are always fatal there; they are called miliary from their resemblance to millet seed in size, and also in color, shape, and appearance.

It is impossible to examine the lungs of fifty grown persons who have died suddenly, with or without any common sickness, after living fifty years, and not find absolute demonstration of two facts: that half of them have more or less of tubercles in the lungs, and that a quarter of them have had more or less a decay, a consumption, or ulceration of the lungs, which had healed up, leaving a scar, just as a bleeding, or a vaccination, or a cut leaves a scar; and as the scar could not have existed without the cut or wound on an arm, so it could not possibly be present in the lungs unless there had been some sore or other ulcerous decay; the cure of this consuming process, this arrest of consumptive decay, having been effected spontaneously by Nature's operations.

Whether art or man can effect this arrest of decay, and can cure, is another thing. Consumption consists in —

First. A want of breath.

Second. A want of flesh.

Third. A want of strength.

Fourth. An everlasting fever, which is all the time burning up the man, proven by a pulse always over the nineties. Cough and expectoration, although nearly always present, are not essential, for the lungs have been found to be nearly half gone, without either ever having been noticed. If you can supplement the breath, and flesh, and strength, and subdue the pulse, to such an extent will the malady be arrested and the man be cured.

Can these things be done, and if so, how?

A new cure for consumption comes out almost every year; it has its brief day, and passes from the sight and memory of men. Up to this time, no medicine has been discovered which has any appreciable effect in even arresting, materially, the steady progress of the disease; and yet men and women and children do get well of consumption every year, and eventually die of some other disease, demonstrated by the actual examination of the lungs after death. It was stated in the "New York Herald," at the time of General Jackson's death, that, on an examination, one third of the lungs had decayed away, referable to a state of things which existed thirty years before.

It is known that pregnancy arrests consumption in many cases, the arrest continuing until the child-bearing period has passed, and sometimes to the close of life. If consumptive persons become asthmatic, the disease is displaced for life.

Consumption is often permanently arrested by the spontaneous occurrence of running sores in any part of the body, or by extensive rashes making their appearance on the skin, and remaining there. At the same time, it should be borne in mind that artificial sores or runnings never cure, but rather hasten a fatal termination by their debilitating effect. Sometimes consumption is cured by a sudden and marked change of mode of life, such as sedentary persons, those who have lived in-doors mainly, taking some occupation which compels a large exposure to the open air, night and day, regardless of all weathers; a compulsory exposure, such as a berth before the mast for a three or four years' whaling voyage in far north latitudes; or driving cattle; or in long exploring expeditions by land or sea, where it is impossible to sleep under a roof, except occasionally, with frequent living on short allowance of food, and occasional danger of starvation. Now, why is all this? Let the reader think for himself a moment, for every one is personally interested in the matter. It is absolutely certain that the reader of these lines will die of consumption, or some very near friend or relative. The very essence of consumption is failing breath, and failing strength and flesh; these are always present. We all know that flesh and strength depend altogether on a good digestion. Then again, if the breath fails, that is, there is shortness of breath, it is because air enough does not get into the lungs; hence what does get there should be the purest air possible, and the most of it possible, every breath of it, if it can be brought about. It then indubitably follows that the two conditions requisite to the cure of consumption are —

A vigorous digestion.

An out-door life, — a hunter's life, for example, very many miles away from all human habitations, being compelled to live on what could be caught, or hunted, or gathered, as birds, animals, fish, berries, and roots, sometimes having nothing to eat for a day or two; or if there be plenty of food, there should be compulsory exposure to all sorts of weather, as before the mast.

A CASE.

There was, forty years ago, a New England family of six or eight sons and daughters. Most of them died at maturity, of consumption, and with pretty much the same symptoms. The youngest boy concluded, at eighteen, that he would likely follow those who went before him. He at once went far south, to the unbroken wilds of Arkansas, and followed the life of a hunter and trapper for a number of years, seldom coming into the "settlements." He grew to be a man weighing two hundred pounds, studied law, and lives to this day as a famous general of the war. Out-door air, out-door effort, then, give a vigorous digestion, and root out consumption, even in advanced stages, as in General Jackson's case.

See the extraordinary case of Norcomb, in our book on "Coughs and Colds," published by Hurd and Houghton, New York, and others in our last book on "Health at Home."

